# Mechanistic intersections between migraine and major depressive disorder

**DOI:** 10.1186/s10194-025-02097-x

**Published:** 2025-07-09

**Authors:** Micah Johnson, Yassine Filali, Anjayooluwa Adegboyo, Maureen Eberle, Abigail Townsend, Yamam Hussien, Catherine Marcinkiewcz, Rainbo Hultman

**Affiliations:** 1 Department of Molecular Physiology and Biophysics, Iowa Neuroscience Institute, University of Iowa, Iowa City, IA USA; 2 Interdisciplinary Graduate Program in Neuroscience, University of Iowa, Iowa City, IA USA; 3 University of Maryland Baltimore County, Baltimore, MD USA; 4 Department of Cellular and Systems Pharmacology, University of Florida, Gainesville, FL 32610 USA; 5 Department of Psychiatry, University of Iowa, Iowa City, IA USA

**Keywords:** Migraine, Major depressive disorder (MDD), Stress, Neuromodulators, Neural circuits, Sensory processing, Emotion

## Abstract

It has long been recognized that there are comorbidities between migraine and psychiatric disorders, especially major depressive disorder (MDD). Often this relationship is attributed to the psychological risk of having an intermittent debilitating disorder that causes a great deal of suffering. However, as evidence mounts about the mechanisms underlying each disorder, mechanistic overlap is beginning to emerge that suggests some shared underlying etiology. These findings span biochemical, genetic, physiological, neuroanatomical, and environmental levels of analysis. Here we review evidence for shared biological mechanisms between migraine and MDD by first searching for mechanisms of each disorder separately across levels of analysis, with an emphasis on current and historic treatments for each disorder. Search terms included each disorder separately largely across NIMH Research Domain Criteria (RDoC) levels of analysis, adding “environment” to genes, molecules, cells, circuits, and physiology. In order to focus our efforts on shared underlying mechanisms, we included studies in preclinical models of these disorders. This review was written to highlight the emergent themes with the most mechanistic overlap between the two disorders across levels of analysis. We prioritized reporting of the findings with more mechanistic detail, which often comes from preclinical studies with carefully controlled variables and tests of causality. As such, this review is aimed at providing new insight by identifying features that are shared between these disorders, including those that have largely not been previously recognized. By emphasizing preclinical studies and mechanisms of underlying *constructs* relating these disorders, such as sensory gain and processing of emotional stimuli, we intend for this review to stimulate new areas of investigation at both clinical and preclinical levels of research.

**Supplementary Information:**

The online version contains supplementary material available at 10.1186/s10194-025-02097-x.

## Background

Migraine and Major Depressive Disorder (MDD), while presenting with very different types and timing of symptoms, are highly and bidirectionally comorbid [1, 2]. Patients with MDD are 2–3 times more likely to have a migraine diagnosis and patients with migraine have a ~ 2-fold increased likelihood of a depression diagnosis [3–5].

The International Classification of Headache Disorders 3rd Edition classifies migraine into nine different categories that all must include at least five attacks with the following attributes:

1. Headache lasting 4–72 h (untreated),
2. Unilateral, pulsating, moderate/severe, or aggravated by physical activity (must have 2/4), and
3. Nausea/vomiting and/or photophobia/phonophobia [6].

In the United States, major depressive disorder (MDD) is defined by the Diagnostics and Statistical Manual (DSM) V as patients having five of nine different criteria spanning symptoms [7]:

1. Depressed mood,
2. Diminished interest or pleasure,
3. Significant changes in appetite or weight,
4. Sleep disturbances,
5. Psychomotor agitation or retardation,
6. Fatigue,
7. Feelings of worthlessness or excessive guilt,
8. Diminished ability to think or concentrate,
9. Recurrent thoughts of death or suicide [7].

Both disorders disproportionately impact women and girls with migraine having a 3:1 female to male ratio [8, 9] and depression having a 1.5-2:1 female to male ratio [10, 11]. Depression and migraine have been reported as the leading and second-leading causes of disability worldwide, respectively [12–15].

Both disorders display a high degree of heterogeneity in presentation, making the search for underlying etiology especially challenging. Migraine episodes commonly occur in response to “triggers” which vary among individuals and encompass a diverse array of factors, ranging from stress and hormonal fluctuations to weather changes, specific sensory stimuli (odors, lighting, sounds), exercise, and diet [16]. These triggers play a significant role in precipitating episodic migraine attacks but can be unreliable predictors and are often unavoidable [16]. Migraine also involves multiple phases, several of which include fluctuations and predictable disruptions in mood [17]. In addition to the headache attack phase, which is characterized by severe pain, nausea, and sensory hypersensitivity, the prodromal and postdromal phases last up to 48 h before or after headache pain, respectively. The phases include common symptoms of fatigue, repetitive yawning, nausea, difficulty concentrating, and, especially during prodrome, pallor and predictable disruptions in mood [6, 15]. Migraine with aura is a subtype of migraine, occurring in about 20% of migraine patients, characterized by an additional phase that includes transient focal neurological visual, sensory or other central nervous system symptoms that usually precede headache [6].

Major depressive disorder is also a complex and heterogenous disorder with a wide degree of variance in its clinical presentation, risk factors, and therapeutic responses among patients [18, 19]. Altogether, these highly comorbid disorders likely both have a more diverse array of underlying causes than is currently appreciated. While we have yet to fully elucidate the pathogenesis of either disorder, there is a growing body of literature indicating shared underlying mechanisms between them. Here we reviewed common overlapping mechanisms contributing to these two disorders. Because there is a large body of data and often the most clearly interpretable mechanistic data comes from preclinical models of these disorders where carefully controlled laboratory conditions can get closer to establishing causality, we emphasize such studies. The prevalence of both migraine and MDD in women and girls compared to men and boys would seem to suggest possible sex-dependent mechanisms, however, many of the studies reported here identified mechanisms present in both sexes. The comorbid condition also appears to be more common in female patients, however, more studies are necessary to precisely understand this comorbid population [20]. We make specific reference when studies found differences in males and females for specific mechanisms. Finally, in order to increase accessibility to a broad audience, we have organized our description of this evidence across levels of analysis starting with genes and moving up in levels of analysis across molecules (genetics, gene expression, and pharmacological treatments), cells (neuromodulation and neural circuitry), physiology (functional neuroanatomy, gut and whole-organism stress responses) and environment (stressors and gut-brain axis).

### Genetics & gene expression

Migraine and MDD are both polygenic disorders for which there is rich body of data indicating a shared genetic link [21–23]. Given that this genetic link has been reviewed extensively elsewhere [21–25], we will keep our summary brief and limited to a history of major key points that form the foundation of the mechanistic links between these disorders. Twin and family studies have indicated that genetics plays a major role in the shared comorbidity between migraine and MDD [24]providing a strong foundational starting point for examining intertwining mechanisms across levels of analysis [23]. Building on this work, a recent comparison of GWAS studies identified migraine as the only neurological disorder to have a shared heritability with psychiatric disorders, with MDD being the strongest [22]. Genome-wide association studies (GWAS) of both depression and migraine continue to reveal commonalities in genetic risk factors for the two [26–30].

Further in-depth evaluation of GWAS network studies have identified key genetic risk variants and genes common to both disorders [25]. After considering broadly defined gene-based evidence for association and accounting for 13,524 gene-based tests, two genes especially stood out as meeting the most stringent statistical criteria for genetic features shared between MDD and migraine, ANKDD1B and KCNK5 [25, 31]. ANKDD1B, ankyrin repeat and death domain containing 1B, is linked to the coding of ankyrin-repeat proteins, which are involved in several human diseases, especially migraine and has elevated expression in microglia [25, 32, 33]. KCNK5, which encodes a potassium channel, is a target candidate for central nervous system illnesses due to its association with migraine and depression sensitivity via related protein family members such as KCNK18 or KCNK4 [25, 34]. This study further identified three novel single nucleotide polymorphism (SNP) loci associated with both disorders, with current evidence supporting a biological role in migraine or MDD for three of these candidate genes. CDCA2, Cell-division cycle associated 2, is involved in pathways which overlap across migraine with aura and without aura [25]; EBF2, Early B-cell factor 2, regulates dopaminergic neurons in the midbrain periaqueductal grey matter, which suggests pain modulatory impact [25]; and RPLP1, ribosomal protein lateral stalk subunit P1, involved in ribosome pathways of protein synthesis/degradation, has been shown to be related to MDD in a mouse model [20, 35].

A recent study evaluating overlapping gene expression altered in both MDD and migraine identified four cell types with common gene expression patterns between the two, including microglia, astrocytes, and neurons within the CNS and peptidergic nociceptors in the PNS that express CGRP and Substance P [36]. This study further identified eight functional categories of overlapping gene expression between the two disorders [36]. These functional groups include: synthesis/binding of neuropeptides, dopaminergic and serotonergic neurotransmitters, glutamatergic neurotransmitters and endocannabinoid genes, calcium channel, inflammatory factors and immune responses, hormonal genes, vascular regulation, and drug metabolism [36]. As it has been genetic studies that formed the basis for the first mechanistic linkages identified between migraine and MDD, it is perhaps unsurprising that these categories show up repeatedly when considering shared mechanisms at other levels of analysis, which we review here.

### Pharmacology & therapeutics

Clues to possible shared mechanisms underlying these disorders stem from overlapping mechanisms of their treatments. Migraine therapies [37] include both acute abortives such as non-steroidal anti-inflammatory drugs (NSAIDS), triptans [38] and ditans [39]CGRP antagonists [40]as well as long-term prophylactic treatments such as beta-blockers, anticonvulsants, antidepressants, calcium channel blockers [41]botulinum toxin injections [42]and anti-CGRP antibody injections [43]. Non-pharmacologic treatments for migraine include diet, acupuncture, neuromodulation (both peripheral and central), biofeedback, and other lifestyle adjustments [44]. First-line therapies for depression includes selective serotonin reuptake inhibitors (SSRIs), tricyclics, other monoamine neuromodulator pharmacologics and psychotherapy [45, 46]. Treatment-resistant depression therapies include ketamine, psilocybin, electroconvulsive therapy (ECT), transcranial magnetic stimulation (TMS), and, experimentally, deep brain stimulation (DBS) [46, 47].

Drugs that regulate serotonin (5-HT) pathways or increase serotonin’s synaptic duration or other migraine and MDD-associated neuromodulators have been demonstrated to be effective in treating some patients for both chronic migraine and depression [48–50]. In the case of acute migraine treatment, the triptans and ditans are serotonin agonists with affinity to receptors 5-HT1B, D, and F that reduce symptoms of migraine including hypersensitivity, cortical spreading depression (CSD), and vasodilation during migraine attacks [38, 39, 51]. These drugs are thought to work by activating presynaptic 5-HT1B/D/F receptors on cerebral calcitonin gene-related peptide (CGRP) terminals and inhibiting its release to promote vasoconstriction although accumulating evidence suggests that there may also be central mechanisms of action that could inhibit release of other neurotransmitters [52–54]. Interestingly, 5-HT1B receptors have also been implicated in MDD [55, 56]. Genetic knockout studies in mouse models have reported that total ablation of the 5HT1B receptor has an antidepressant-like effect [57–59]. Another class of drugs that has shown efficacy in preventing migraine and treating depression are the tricyclic antidepressants (TCAs) and serotonin-norepinephrine reuptake inhibitors (SNRIs), which can potentiate the actions of serotonin and norepinephrine action by inhibiting reuptake via their respective transporters, the serotonin transporter (SERT) and the norepinephrine transporter (NET). Amitriptyline (a TCA) and venlafaxine (an SNRI) are antidepressants in each class that have demonstrated successful migraine prevention [60–62]. These drugs tend to increase serotonergic transmission more broadly across the brain, so it can be difficult to pinpoint any particular brain region or pathway that is mediating these effects. However, other serotonergic compounds have dual anti-migraine and antidepressant effects including psilocybin, a psychoactive substance with hallucinogenic properties that acts as an agonist at 5-HT2A receptors [63–67]. Together, these studies provide converging evidence that migraine and MDD may both exhibit therapeutic responses to serotonergic drugs. While sex-specific differences in responses to these drugs requires further study, side effects of serotonergic drugs tend to be worse in women than men and serotonin is known to be regulated by sex-specific hormones such as estrogen, to be metabolized differently in women and men, and to have sex-specific downstream effects such as on the hormone prolactin [68].

Episodic onset of both migraine and MDD are associated with the dysfunction of signature neuropeptides. In the case of migraine, elevated levels of CGRP is a target for treatments that abort and/or prevent migraine in some patients [40, 69]. CGRP exists in two isoforms (α and β) and is widely expressed in sensory neurons, motor neurons, and neuroendocrine cells throughout the body. CGRP is known to act as a potent vasodilator and plays multifaceted roles in various physiological systems. Its actions are mediated through G protein-coupled receptors that form complexes with receptor activity-modifying proteins, enabling diverse physiological functions across various systems. Elevated levels of CGRP are observed during migraine attacks, and its infusion has been shown to trigger migraine-like symptoms [69]. Interestingly, CGRP levels have been found to be even higher in migraine patients with depressive symptoms than in those without [70]. Monoclonal antibodies targeting CGRP or its receptors have been demonstrated not only to reduce the number of migraine days per month by about half, but also to mitigate depressive symptoms in patients with the comorbidity as well [71–73]. There is much that remains to be investigated regarding the context, frequency, and mechanisms of these findings but one early longitudinal study found that reduction in depressive symptoms was independent of headache frequency [74]. In MDD, increased neuropeptide Y (NPY) activity has been shown to have an antidepressant effect [75–77]. Although the mechanisms of how these neuropeptides modulate each disorder are not fully understood, it has been demonstrated that intraperitoneal injection of CGRP downregulates NPY expression in rat hypothalamus, suggesting that CGRP may reduce NPY’s bioactivity [78, 79].

Another important peptide implicated in both migraine and MDD pathophysiology is PACAP (pituitary adenylate cyclase-activating polypeptide). PACAP-related signaling and expression within the CNS is associated with neural circuits related to pain, stress, depression, and related functional domains [80]. At the time of writing of this review, it is a target in phase II clinical trials for migraine and has been linked to the biology of most major therapeutic strategies in depression [81, 82]. Like CGRP, PACAP’s peripheral mechanisms include being a vasodilatory peptide that is elevated in migraine and causal in stimulating migraine responses in both humans and rodents; both preclinical and clinical trials suggest likelihood of future therapeutic success in migraine and especially for some of the more complex feature of migraine, including chronic and medication overuse headache [83–85].

PACAP has also been shown to play a role in mood disorders related to stress, especially MDD, with hypothalamus, amygdala and prefrontal cortex playing an especially important role [80, 86, 87]. PACAP expression is elevated in some brain regions in MDD [86, 88]. Substantially higher PACAP expression levels were found across sexes in the dorsolateral prefrontal cortex (dlPFC) and anterior cingulate cortex (ACC) of patients with MDD compared with controls [86]and in the central bed nucleus of the stria terminalis (cBNST) specifically in men but not in women [88]. Single nucleotide polymorphisms (SNPs) in PACAP have been found to be more prevalent in MDD across sexes, though it is unclear what impact this has on PACAP expression or function [89]. PACAP expression in the absence of disease state differs between the sexes, with PACAP more highly expressed in the PVN in females compared with males [80, 90]. While correlation does not indicate causality, these findings, as well as estrogen’s interaction with PACAP signaling, at baseline and in response to stress, make PACAP signaling a possible candidate for a mechanism of sex dimorphism in migraine and MDD [80, 91]. Estrogen’s impact on PACAP is not perfectly straightforward, but does play some role as evidenced by changes in PACAP levels in women at different menstrual cycle stages and/or with regulated estrogen levels. Overall, PACAP MDD data in humans to date is correlational, which does not disambiguate whether PACAP is causal toward MDD. More studies are needed to determine whether this relationship is causal or compensatory.

Many preclinical studies have attempted to investigate a causal relationship between PACAP and depressive responses [80]. Caution ought to be taken in interpreting certain behaviors in animal models as “depressive,” such as immobility on the tail suspension test or forced swim test. That said, PACAP has been shown to elicit a rapid reversal of such phenotypes (i.e. PACAP has antidepressant-like response) globally, and in certain brain regions (hippocampus, prefrontal cortex) in preclinical models such as chronic social defeat stress and lipopolysaccharide exposure [92, 93]. PACAP has even been shown to be necessary for the therapeutic effects of antidepressants such as paroxetine, ketamine, and zhizichi decoction [92, 93]. Similar experiments using global systemic knockouts of PACAP resulted in depressive-like phenotypes, suggesting PACAP to have an anti-depressive effect on a systemic level [94]. PACAP signaling has also been implicated in the appearance of pro-depressive behaviors across several key brain regions, including the PVN and central amygdala (CeA) [95, 96]. Given the complexity of PACAP's intersection with mood, we provide Table S1 as an overview. A large body of data also points toward an intersection between PACAP and the hypothalamic-pituitary-adrenal (HPA)-axis, which we revisit in the environmental stressors section below [97]. More ongoing studies are necessary to better understand the precise localization and mechanistic context in which PACAP contributes to both migraine and MDD, especially in preclinical models that test sex differrences; much of the work to date has been done only evaluated male rodent models (Table S1). Currently, systemic manipulations of PACAP seem more promising on a migraine front, whereas more nuanced spatial regulation and HPA-axis engagement appears necessary to consider with regard to depression. Overall, a substantial body of PACAP data suggests that anti-PACAP migraine therapies should be approached carefully and studied well for those with co-morbid MDD. Depending on the physiological trajectory upon administration, there is a potential to exacerbate (or alleviate) depressive symptoms.

Another pharmacological agent that points toward possible linkages between migraine and depression is topiramate, an anticonvulsant prescribed for migraine prophylaxis [98]. The impact of topiramate on mood is complex, with effects that range from mood stabilizing in patients with bipolar disorder to pro- or anti-depressant [99–102]. As a prophylactic treatment for migraine, approximately 10% of topiramate-treated patients experience depression, and depression risk increases significantly in patients with a history of depression [103, 104]. One possible mechanistic explanation for topiramate’s role in migraine and depressive side effects includes its reduction of excitatory neurotransmitter release [105]. With regard to migraine, this is thought to prevent release of vasoactive peptides including CGRP, promoting inhibitory GABA neurotransmission [105]. Additionally, in a rat model of migraine, chronic administration of topiramate was shown to suppress cortical spreading depression [106]. GABA/Glutamatergic balance appears to be dysregulated in both migraine and MDD; elevated levels of Glutamate can be found in blood and CSF in both migraine and MDD [107–109] and at least one study identified decreased levels of GABA in chronic migraine patient CSF, with even lower levels in patients with the comorbid condition [110].

A review of randomized controlled trials in patients that used topiramate for the treatment of migraine found that, while topiramate was effective in reducing migraine days per month compared to placebo, there was a consistently high number of adverse effects which led to treatment discontinuation throughout these studies and especially with higher treatment doses (≥100 mg/day). Such adverse effects included paresthesia, anorexia, weight loss, fatigue, nausea, and cognitive and memory problems [105]. Notably, several of these are also symptoms of or are related to MDD. The inhibitory mechanisms of topiramate’s prophylactic effects in migraine may explain its depressive side effects, which also appear to be dose- and dose-titration dependent [103, 104]. Most studies detailing the depressive side effects of topiramate are epilepsy studies, which indicate that these are more frequent in individuals with a personal or family history of psychiatric disorders or with more severe forms of epilepsy [103, 111]. Perhaps contrarily, in female patients with only moderate recurrent MDD, a study of *n* ~ 60 observed that topiramate monotherapy was shown to be effective in reducing depression severity over time, and though significant weight loss was associated with topiramate, the study found no severe side effects [99]. In patients with treatment-resistant MDD, adjunctive topiramate with SSRIs was shown to reduce depressed mood, suicidality, insomnia, agitation and anxiety symptoms [101]. Both of these studies were small < 100 subjects and require larger double-blind studies before conclusions could be drawn. Furthermore, there were no distinct clinical or demographic features associated with a reduction in depressive symptoms during topiramate treatment in the latter study. Studies delineating patient-specific factors (e.g., dose, comorbidities, genetic polymorphisms) that may predict anti-depressant or depressant response to topiramate would be required to untangle the complexities of topiramate in MDD and migraine. Overall, topiramate may be a useful treatment for moderate treatment-resistant MDD or migraine individually, but it may be best reserved for those with a mono-diagnosis and mood symptoms should be carefully monitored regardless of diagnosis. Given the heterogeneity of side effects and outcomes, it seems unlikely to ever become a first-line therapeutic for either disorder, and it seems likely that for most patients, the potential costs outweigh the benefits, but it does provide some interesting clues to mechanisms involved in each, especially related to excitatory/inhibitory balance [111].

Ketamine is an antagonist of N-methyl-D-aspartate (NMDA) receptors that has shown some success in treating both migraine and MDD [112, 113]. In MDD and bipolar depression, ketamine provides a rapid and robust antidepressant effect with an onset of 40 min after a single intravenous infusion [112]. Although maximum efficacy is seen at 24-hr post-administration, this effect on depression is transient, disappearing 1–2 weeks post-infusion [112, 114]. In migraine, ketamine has shown efficacy for patients across ~ 12 studies, most studies testing treatment refractory migraine, with several studies focusing on familial hemiplegic migraine, and a few focusing on chronic migraine [113, 115–117]. In one study, intravenous ketamine infusion resulted in reduction in headache pain (reduction verbal pain rating scale) in nearly three-quarters of chronic migraine patients [116]. Several studies demonstrated intranasal ketamine infusion reduced pain scores in patients with refractory migraine (reduction in numeric rating scale for pain intensity) [116, 117] or familial hemiplegic migraine aura [118, 119]. However, it is worth mentioning that many of the clinical studies involving ketamine in the treatment of migraine are limited (ranging from 6 to ~ 150 patients) and further studies with adequate controls and sample sizes are needed [115, 120]. While no studies to date have explicitly studied whether efficacy is greater in the comorbid migraine/MDD condition, several of the studies of the efficacy of ketamine for treatment-refractory migraine or headache included substantial percentages of patients with the comorbidity, with one (Yuan et al.) reporting slightly higher numbers of the patients with ketamine success in the comorbid condition [116, 117]. Of all studies reported to date, primary side effects were temporary blurred/double vision, hallucinations, and confusion, however, one patient in a single study of IV-infused ketamine did experience suicidal ideation [116]. Overall, ketamine may be a good option, especially via intranasal or subcutaneous routes of delivery, for treatment refractory migraine in the context of MDD, under careful observation. Although the exact mechanism of ketamine’s therapeutic effects is not completely known, beyond its role blocking NMDA receptors, ketamine is known to inhibit nitric oxide production and block calcium and sodium channel signaling, all of which are causal mechanisms in migraine [112]. Moreover, preclinical evidence has shown that ketamine displays other mechanisms of action that may provide insights into shared mechanisms between migraine and MDD involving actions at 5HT1B receptors and 5HT transporters, increasing 5HT, GABAA, and nicotinic acetylcholine, and increasing expression of hyperpolarization-activated cyclic nucleotide-gated (HCN) channels, specifically, the HCN1 receptors [121–123]. More recent studies demonstrate an anti-inflammatory role for ketamine as well, which has implications for both migraine and MDD [124].

Hormonal regulation is more common with regard to treatment or prevention of migraine than mood disorders, however, there is ample evidence of estrogen’s relationship to MDD as well, which we discuss more in the section on stress below [125–127]. Thus, hormonal regulation of estrogen is sometimes used in the treatment of the comorbid condition in women [128].

Finally, B vitamins have also been suggested to play a role in both disorders. Vitamin B2 (riboflavin) is effective in treating chronic migraine, and some MDD patients have been shown to have vitamin B2 deficits [129, 130]. Higher dietary vitamin B2 intake is associated with lower severity of chronic migraine and MDD, a correlation that appears to be more significant in female populations [129, 131, 132]. Daily riboflavin needs can vary by life stage and sex [133]. While there is more to be investigated about the underlying mechanisms by which riboflavin exerts its effect on these disorders, mitochondrial activity has been implicated as a possible route for further investigation [132, 134].

Taken together, what’s known about the therapeutic treatments for both migraine and MDD give us important clues to the underlying mechanisms that contribute to each disorder and mechanisms that maybe apply to both, or the comorbid condition. There are a number of classes of pharmacological treatments in particular that should be especially considered with regard to individuals demonstrating comorbidities between these two disorders. Regulation of neuromodulators is an especially important recurring theme that emerged and as such, we will further delve into what is known about these mechanisms of in the following section.

### Monoamine neuromodulators

The data regarding overlapping genes and treatments of MDD and migraine point us toward neuromodulators as important to the molecular and cellular mechanisms shared between these disorders. It has been recognized for many years that aberrant regulation of serotonin, dopamine, and monoamine regulation more broadly is important in migraine and MDD [135]. Disruptions in aminergic neurons, particularly serotonin, dopamine, and norepinephrine systems, alter the regulation of mood and pain perception [50]. Importantly, sex differences in monoamine regulation have been identified for both MDD and migraine [127, 136].

Dopamine is a neuromodulator that is most commonly associated with reward and motivation, which has an established role in depression and for which there is an evolving appreciation of its role in migraine as well. One of the earliest identified common mechanistic links on this front is the involvement of dopamine receptor, DRD2, which provided one of the first genetic linkages between migraine and MDD [137]. In particular, a significant association exists between genetic variations in the dopamine D2 receptor (DRD2) gene and increased susceptibility to migraine with aura (MWA) and major depression [137]. DRD2 is known for its functional selectivity and role as a therapeutic target in psychological and neurological disorders. DRD2 predominantly acts as a presynaptic autoreceptor, which suggests involvement in the regulation of neurotransmitter release, possibly influencing migraine pathogenesis [138]. Similarly, in MDD, dysregulation of dopamine neurotransmission is implicated in the neurobiology of the illness [139]. More recent studies in rodent models of migraine have further explored a mechanistic role of DRD2 using receptor agonist and antagonists; DRD2 antagonism reversed acute migraine-response metrics including allodynia, CGRP levels, and brainstem c-fos activation [140]. Moreover, the DRD2 agonist associated with migraine relief has been mechanistically linked to the regulation of AMPA receptor trafficking via a PI3K, Src family kinases (SFKs), pathway in a rat model of migraine [141].

Natural DRD2 ligands, including dopamine and trace amines like tyramine, exert influence on DRD2 function, potentially impacting neurotransmitter release and neuronal excitability relevant to migraine pathogenesis and depressive symptoms. Conversely, synthetic ligands offer targeted interventions: antipsychotics such as risperidone and haloperidol antagonize DRD2, providing relief from psychotic symptoms in some cases, while dopamine agonists like bromocriptine and pramipexole hold promise in managing depressive symptoms or associated migraine features by stimulating DRD2 activity [138, 139]. In migraine, dopamine has been implicated as playing a role clinically, hormonally, and genetically across multiple phases of migraine, including interictal periods [142, 143]. Specifically, there is a decrease in endogenous dopamine release in the striatum during the ictal phase (migraine attack) and allodynia in migraine patients compared to healthy controls, suggesting a dopamine-related mechanism for headache pain and sensory hypersensitivity during migraine attacks [142]. Interestingly, another study using PET imaging found that interictal endogenous DRD2/D3 availability in the nucleus accumbens of episodic migraine patients is inversely correlated with positive affect, which may explain the underlying emotional aspect of pain in migraine [144]. Interestingly, patients with depression showed greater D2/3 receptor availability in several striatal regions, including the bilateral ventral pallidum/nucleus accumbens (vPAL/NAc) [145]. Further, the localization of dopamine receptors within the trigeminovascular system (blood vessels associated with the trigeminal nerve) also suggest a role of dopamine signaling in headache pain [142, 146]. As mentioned above in the genetics section, a study investigating shared risk genes across different cell types in migraine and MDD found that dopaminergic and serotonergic neurotransmitters were present throughout the peripheral and central nervous systems [36]. Notably, genes related to dopamine were highly expressed in peripheral sensory receptor cells: peptidergic and non-peptidergic nociceptors, large diameter neurofilament-positive mechanoreceptors, and C low threshold mechanoreceptors, suggesting that dopaminergic dysregulation in these cells could be linked to migraine with comorbid depression [36]. Subtle sex differences in D2R availability has been identified via PET imaging, including for seasonal variations over time, with male subjects having lower D2R availability [147].

Norepinephrine plays a crucial role in concentration, memory, attention, arousal, sleep, and transmission of pain signals from the meninges [148, 149]. Norepinephrine’s influence on these cells increases their responsiveness to pain and triggers the release of inflammatory substances like interleukin-6 (IL-6), which potentiates pain signaling [149]. It has long been recognized that norepinephrine plays a pivotal role in MDD [150]supported by the efficacy of serotonin and noradrenergic reuptake inhibitors (SNRIs) in treating symptoms of depression and chronic pain conditions [60]. As mentioned above, SNRI’s also have efficacy in migraine prophylaxis as well [151–153]. The locus coeruleus-norepinephrine (LC-NE) system has been demonstrated in preclinical studies to mitigate nitroglycerin (NTG)-induced migraine-like headache via sleep disruption [154]. Both migraine and MDD are disorders in which sleep regulation is critical to preventing exacerbation of the disorder and its symptoms [155–157]. Differences in LC size, cell composition, and regulation of NE occur between sexes, with estrogen an important regulation of NE, making this another possible point of sex dimorphisms in migraine and MDD [158, 159].

Serotonin is probably the neuromodulator most widely linked to both MDD and migraine and has to some extent been reviewed well previously [135, 160]. Serotonin plays a crucial role in various physiological processes, including mood regulation, pain perception, and vascular function, and is thought to promote emotional well-being and positive affect. While the precise role of serotonin in MDD has been questioned in a recent meta-analysis [161] others report that reduced serotonin activity may nonetheless play a role in the pathogenesis of a subtype of depression characterized by negative emotions, agitation and anxiety [162] and that the role of serotonin in the etiology of MDD is complex and dynamic [163, 164]. This view is based upon substantial biochemical and metabolic neuroimaging studies across a diverse group of MDD patients. Neuroimaging and autoradiography studies indicate a relationship between depression severity and reductions in serotonin transporter (SERT) binding in the amygdala [165] and neocortex [166] while 5-HT1A receptors are elevated which leads to reduced serotonin neuronal firing and release in the cortex [167, 168]. Thus, the observation that tryptophan hydroxylase 2 (Tph2), the rate limiting enzyme in serotonin synthesis, is increased in the raphe nuclei of depressed suicides may represent an attempt to compensate for serotonin hypofunction [169, 170]. Finally, there are compelling studies indicating that tryptophan depletion, which lowers serotonin biosynthesis, is a potent trigger of depressive episodes in a subset of depressed patients [171, 172].

Likewise, dysregulation of serotonin systems has also been implicated in migraine pathogenesis [173, 174]. The intricate serotonin system comprises numerous receptor subtypes, including those targeted by migraine drugs such as triptans, each with distinct functions and distributions across the central nervous system and other tissues. In migraine, serotonin is believed to play a dual role, acting both as a vasoconstrictor and a neuromodulator. Fluctuations in serotonin levels have been associated with the onset and progression of migraine attacks, with serotonin depletion often preceding the headache phase [175]. Additionally, serotonin is involved in pain processing pathways, influencing the perception and severity of migraine pain [176]. Dysfunction in downstream serotonin signaling pathways, including alterations in receptor sensitivity and neurotransmitter release, has been specifically linked to the pathophysiology of migraine [176]. Studies have demonstrated that decreased levels of serotonin are associated with migraine attacks, suggesting a crucial role for serotonin in modulating pain pathways and vasodilation of blood vessels implicated in migraine headaches [176]. There is also an interesting link between genetic polymorphisms that alter promoter activity in the 5-HT2C receptor and migraine in a Turkish population [177, 178]. 5-HT2C receptors have also been shown to modulate dopamine release and are linked to the therapeutic efficacy of antidepressants [179].

The intersecting roles of neuromodulators in MDD and migraine would be incomplete without at least mentioning evidence supporting the involvement of the gut-brain axis [180–183]. Clinical and preclinical studies have linked populations of microbiota to both migraine (Firmicutes Clostridium; Helicobacter Pylori, negative association with Agathobacter) [184–186] and MDD (Firmicutes and Bacteroidetes, Enterobacteriaceae and Alistipes, negative association with Lachnospiraceae) [187–189]. Altered 5-HT regulation is reflected in fecal microbiota transplantation studies in which transferring the microbiome of MDD patients to rodents has been shown to induce depressive-like behaviors in the recipients [190]. Kang et al. showed that susceptibility to NTG-induced migraine-like pain was also transmissible from a migraine patient to mice via fecal microbiota transplantation, which was not seen in mice treated with the microbiota of healthy controls [191]. Probiotic treatment has been shown to ameliorate depressive-like behaviors in human and rodent models of stress and depression, possibly due to these probiotics increasing levels of the serotonin precursor tryptophan and consequently increasing 5-HT availability [192].

### Functional neuroanatomy & neural circuitry

An integral component of the mechanistic relationship between migraine and MDD lies within their similarities in neuroanatomical and functional characteristics identified across the central nervous system (CNS). More specifically, emerging evidence has shown overlapping impairments in neural architecture and functional activity at the levels of brain regions, neural circuits, and interconnected brain-wide networks; underscoring the neurophysiological basis for the bidirectional nature and convergence of symptomologies. Commonalities in their observed neural pathologies include structural malformations, dysfunctional reactivities, hyper/hypo-connectivity patterns, and impairments in neural network pathways and activities. These shared divergences have been primarily identified through investigations in patients with migraine, MDD, and the comorbid condition, employing various neuroimaging techniques in human patients including positron emission tomography (PET), electroencephalography (EEG), functional magnetic resonance imaging (fMRI), deep brain stimulation (DBS), and transcranial magnetic stimulation (TMS). Furthermore, mechanistic studies in preclinical models, combined with novel neural recording techniques as well as precision circuit and pharmacological manipulations have expanded our understanding of the CNS’s role in the shared manifestation, maintenance, and exacerbation of these disorders [193, 194]. Taken together, the following brain regions have emerged as key candidates at the intersection of migraine and MDD’s pathophysiology: anterior and posterior cingulate cortices [195–197], parabrachial nucleus [198–200], insula [201–203], amygdala [204–207], thalamus [208–211], cerebellum [212, 213], periaqueductal grey [214–217], raphe nuclei, [218, 219] locus coeruleus (LC) [220–222], hypothalamus [223, 224], ventral tegmental area [220, 225], hippocampus [226, 227], nucleus accumbens [228, 229], prefrontal cortices [230–232], and sensory cortices [233–235].

The brainstem is recognized as playing a role in the pathogenesis of both migraine and MDD and may serve as a hub in the pathophysiological overlap of migraine and MDD [236–239]. The brainstem exerts extensive influence on neural circuits and networks that govern arousal, autonomic functions, pain modulation, emotional regulation, and stress response, all of which are shared symptomatic features in these disorders. The brainstem also houses nuclei that serve as sources of neuromodulators (e.g. monoamines) and appear to be differentially activated in both disorders. For example, using transcranial sonography (TCS) Tao et al. found that hypoechogenicity (reduced ability to reflect ultrasound) in the dorsal raphe nucleus is strongly correlated with higher depressive symptomatology in migraine patients and increased migraine attack frequency [218, 240]. Interestingly, this correlation was not observed when comparing migraine patients to control subjects. This hypoechogenicity suggests potential dysfunction in serotonin synthesis, impacting both mood and pain regulation.

Another key player within the brainstem nuclei at the mechanistic intersection is the LC-norepinephrine system. Significant dysregulation in the LC-norepinephrine system occurs in depression, with α-adrenergic receptors (α-ARs) activity playing an important role in aberrant regulation of cognition, mood, pain, and especially arousal, or sleep/wake systems [241, 242]. This is supported by MRI and PET studies that show increased norepinephrine transporter availability in the thalamus of MDD patients, particularly within the thalamic subregions connected to the prefrontal cortex [241, 243]. Within the context of migraine, the LC-norepinephrine system appears to play an important role in trigeminal sensitization and migraine [222, 244]. The LC influences activation of the trigeminal nucleus caudalis, a key structure in nociceptive processing in the context of dural-evoked trigeminovascular activation, through α2-adrenoceptors and noradrenergic projections [222]. Vila-Peuyo et al. demonstrated that disruption of the LC differentially affects migraine phenotypes, where reduced LC activity inhibits dura-evoked trigeminocervical complex activity but increases susceptibility to cortical spreading depression [222]. Critically, the dura-sensitive trigeminal neurons of the trigeminal nucleus caudalis send projections to downstream targets including the thalamus and subsequently to the prefrontal cortex [245]. Functional connectivity of the locus coeruleus is increased in migraine without aura patients [246]. Taken together, dysfunction in the LC may result in abnormal adrenergic modulation along this pathway, potentially explaining the impaired thalamic-prefrontal connectivity seen in both disorders. Thus, these shared disruptions suggest that LC dysfunction could be a central mechanism linking the overlapping pathophysiology of migraine and depression. Notably, studies using rodent models have demonstrated that disruption of the LC can modulate both migraine-related phenotypes and depressive symptoms [222].

Reciprocal connections between brainstem and limbic regions have been heavily implicated in migraine and MDD and their intersection. For instance, individuals with depression exhibit significant decreases in white matter integrity of the right solitary tract, a key afferent pathway from the brainstem to the amygdala [247]. Preclinical trigeminal neuralgia studies indicate that the lateral parabrachial nucleus (LPBN) emerges as a pivotal player in the development of chronic pain and depressive-like behaviors [199]. Glutamatergic projections from the spinal trigeminal subnucleus caudalis (Sp5C) to the LPBN form a direct circuit controlling the emergence of depression-like behavior under chronic neuropathic pain states [199]. Interestingly, head pain in humans has consistently been rated more emotionally taxing than peripheral pain [104]. Rodent behavioral studies provide a mechanism accounting for this effect, which appears to stem from a PBN-amygdala circuit [204, 248]. The CeA more broadly also appears to play a role in the transition from neuropathic pain to depressive states [249].

While migraine is often associated with sensory hypersensitivity and MDD is often associated with negative affect, the neural circuitry across midbrain and limbic regions highlights the important aspects of emotional/affective circuitry in migraine and sensory hypersensitivity in MDD as well. Midbrain and limbic regions, such as the PBN, insula, thalamus, hypothalamus, and amygdala, connect to cortical areas to regulate the emotional/negative affect aspect of pain [250–253]. One study found that nucleus accumbens-amygdala functional connectivity is related to the severity of negative affect in episodic migraine patients [144]. Interictally, migraine patients have demonstrated increased activity measured by fMRI in brain regions related to negative affect specifically in response to negatively valenced emotional stimuli [195]. These data suggest an emergence of a generalized negative affect sensitization across the posterior cingulate, amygdala, caudate, and thalamic regions in migraine [195]. Such sensitization is well-represented in the depression literature, particularly regarding amygdala reactivity in response to negatively valenced stimuli [207, 254, 255]. Reduced connectivity in MDD between the amygdala and frontal regions has been reported across many studies [256]. Mechanistically, optogenetic inhibition of the BLA-ACC circuit can alleviate depressive-like behavior induced by neuropathic pain [257]. There is also a decrease in amygdala to dlPFC resting-state functional connectivity compared between migraine with depression and just migraine patients [258].

Interestingly, sensory processing hypersensitivity, a well-documented characteristic of migraine, has also been found to be increased in MDD patients [259]. This shared sensory processing profile suggests a potential neural mechanism underlying the comorbidity of MDD and migraine, where disruptions in specific brainstem-limbic circuits that process emotive and somatic sensation may be salient. Along these lines, the amygdala, which is altered in both disorders, also plays roles in assigning priority to sensory information from the environment [249, 260].

Importantly, several structural studies have been conducted of amygdala volume in both migraine and MDD. Several studies with ≥ 80 MDD patients have identified functional and structural differences in core nuclei of the amygdala and affective centers in the cerebellum in MDD patients compared with healthy controls [261, 262]. Specifically, the volume of right medial nuclei were larger in MDD while diminished overall gray matter volume was found. One slightly more moderately powered study in migraine patients identified increased bilateral amygdala volume in both patients with migraine alone or migraine with depression [258]. Providing a possible mechanism explaining such alterations, a preclinical study of neuropathic pain suggests a mechanism by which amygdala volume is increased in response to a chronic pain-induced depressive state through cell proliferation resulting in increased neuronal numbers [263]. Taken together, we strongly recommend that future comorbidity studies interrogate amygdala structure with greater numbers. It may be that the ratios of volume and connectivity that can be detected by fMRI in specific amygdalar nuclei could be predictive of a comorbid state.

Higher-order cortical processing is disrupted in migraine and MDD in two important ways: (1) top-down regulation of emotional stimuli is disrupted and (2) attentional bias towards sensory stimuli is heightened. In MDD patients, there is increased activation in the ACC, amygdala, and anterior insula during anticipation and experience of pain, which correlates with perceived helplessness [264]. The heightened attention to both emotional and somatosensory pain is thought to stem from increased activity within the salience network, involving the ACC and insula, which is heightened in both migraine and MDD. Moreover, this network may be hyperconnected via thalamic pathways, wherein functional connectivity analyses have shown increased connectivity between the insula and the posterior thalamus in MDD patients in the anticipation of pain [264]. At the level of top-down modulation, TMS to the dlPFC has been successful in treating migraine, depression, and their comorbidity disease state [265]. Targeting of insular cortex connectivity and salience network appears to explain, at least in part, this response [266]. Data from depressed patients indicates that dlPFC connectivity to striatal regions explains much of the efficacy of this response, suggesting a likely brain-wide network model, which may be the reason for its efficacy in migraine as well [266]. Moreover, several studies have identified functional alterations in the dlPFC and mPFC, as well as in thalamic, temporal, and occipital areas, as distinguishing features of patients with migraine and comorbid depression compared to healthy controls [267, 268]. Collectively, these findings further suggest the hypotheses of dysfunctional dlPFC and thalamic networks governing the comorbidity between migraine and depression.

Lastly, the intersection between migraine and MDD has been linked to structural and functional abnormalities in sensory processing centers including visual, somatosensory, and auditory cortices. Depressed patients display a wide range of sensory abnormalities, which can include higher sensory sensitivity, sensory avoiding behaviors, or low registration to sensory input [259, 269, 270]. Such aberrancies may be due to altered functional connectivity within the visual and auditory cortical networks, an identified feature of clinical symptoms in depressed individuals [271]. Moreover, aberrant sensory hypersensitivity may stem from enhanced network connectivity between the thalamus, limbic, and sensory cortices, which has been observed in MDD [272] and migraine patients [273–275]. Hyper and hypofunction has been observed in somatosensory cortices in both a rodent model and in human patients with depression [276]. The thalamus is a strong candidate as a potential hub contributing to the common feature of sensory hypersensitivity between migraine and MDD with its widespread role as a relay center with functions in pain, sensory, and affective processing, as well as cortical gain mechanisms. Supporting this notion, altered thalamic connectivity is observed in both migraine and MDD, and the comorbid condition, exhibiting enhanced functional connectivity between the thalamic, somatosensory, and insular cortices, suggesting shared dysfunction in sensory processing [211, 268, 272, 275].

### Environmental stressors

Environmental factors, especially stressors of various types, challenge homeostasis and can lead to changes in and outside of the brain that trigger the onset or exacerbation of both diseases, playing an important role in both depression and migraine [277–279]. This has been reported clinically and epidemiologically, but there are also a number of preclinical studies investigating the impact of stress on both depression and migraine, which point to some common underlying mechanisms. Over 60% of the variance in liability to major depressive disorder (MDD) in humans comes from environmental contributions, including stress [280]. Similarly, migraine is also a polygenic disorder with 40–60% likely attributable to non-heritable environmental stimuli [281, 282]. As such, we expect that much can be gained by better understanding how the migraine and depressive brains respond to stress. In the case of migraine, chronic and repeated stress is thought to lower the sensitivity threshold to otherwise innocuous stimuli, triggering migraine attacks [283]. In MDD, chronic stress contributes to the emergence and intensification of the disorder [194, 284]. Investigating brain mechanisms engaged by stress in both migraine and depressive contexts provides an important avenue to consider with regard to shared mechanisms of these disorders.

Clinically, increased adverse childhood experiences are associated with susceptibility to both migraine [285–289] and depression [290–293] in adolescence and adulthood. What we know about the impact of early life adversity on brain development implicates connectivity amongst several key brain regions in these disorders. The amygdala in particular is a brain region whose activity links migraine and depression and has been shown to have aberrant development and connectivity following exposure to early life stress (ELS) [291, 294]. Other regions impacted by increased HPA-axis activity in early life include prefrontal cortex, cerebellum, and hypothalamus, all of which have been implicated in both migraine [212, 294–296] and depression [231, 232, 294, 297, 298]. ELS has also been linked to migraine biomarkers such as endothelial dysfunction involved in inflammation (likely as a result of stress) [287]abnormal HPA axis [299]and immune system dysfunction [300].

Some evidence suggests stress contributes to the higher female prevalence in both diseases [301, 302]. Strikingly, the prevalence of increased headache frequency combined with an adverse childhood experiences score ≥ 5 was three-fold higher in females than in males despite similar population sizes [285, 288]. However, the relationship between increased headache frequency and adverse childhood experiences was statistically significant for both sexes, and the cause of the sex differences is unclear [285, 288]. Researchers debate whether this strongly sex-linked relationship between stress and migraine is a result of females being more likely to report abuse [303], female brain development being more vulnerable to the negative consequences of ELS, or girls and women disproportionally experiencing certain types of abuse (e.g., sexual and emotional abuse) [288].

While the human experience of stress may vary more widely than what is typically modeled in preclinical studies, such studies nonetheless offer a controlled environment to explore the underlying mechanisms. One of the most severe types of ELS in humans is disruption, defined as long periods of separation from the main caregiver. This phenomenon is modeled in different ways in rodents, most often using maternal separation stress paradigms in which rodents are removed from the same cage as their mother at birth [304–306]. In rodents, maternal separation is associated with both migraine-related phenotypes including increased hypersensitivity to light and touch and increased vulnerability to depressive behavior and aberrant brain activity in adulthood [226, 305, 307, 308]. Overall there is more cross-species data on the impact of ELS on brain states related to MDD than there is for migraine, but these depression studies shed light on many of the shared mechanisms we have covered to this point, identifying neural circuit, cell, and molecular changes induced by ELS. ELS in mouse models has been shown to result in widespread gene expression changes and neural circuit engagement associated with depressive brain states across cortico-limbic brain networks including prelimbic and infralimbic cortices, NAc, amygdala, and VTA, dorsal raphe nucleus (DR) and paraventricular nucleus of the hypothalamus (PVN) [306, 309–311]. Serotonin systems are also impacted by ELS, providing yet another connection to the interrelationship between migraine and MDD [312]. Finally, ELS in both humans and rodents alters regulation of inflammatory cytokines, which are known to be important for the emergence of both migraine and MDD [313].

By contrast, only a few preclinical studies have investigated the relationship between ELS and migraine. Rodent models of migraine span the genetic and pharmacological, often using the administration of chemical and biological compounds or stimuli that have been shown to induce migraine in humans to elicit migraine-associate phenotypes such as light-aversion, allodynia, facial grimace, and cortical spreading depression [314, 315]. One study using maternal separation ELS in rats induced chronic migraine by repeated administration of NTG and found that ELS resulted in animals exhibiting differences in climbing, facial rubbing, head scratching, and grooming behavior, indicative of migraine-related phenotypes (e.g., hyperalgesia, allodynia, nociceptive-specific behavior) [316]. Importantly, data from this study also indicated that female rats exhibited more severe migraine-like phenotypes compared to males, supporting the data in humans of ELS more severely impacting girls and women [316]. In a study using C57Bl/6 mice, Eller et al. demonstrated that following administration of NTG, female mice that had undergone maternal separation ELS exhibited increased paw mechanical sensitivity (allodynia), grimace (spontaneous pain), and light aversion that were attenuated by exercise intervention [308]. Both studies found significant differences in migraine-like phenotypes following ELS in female rodents, paralleling findings discussed above in the human literature. Notably, none of these studies address how ELS alters neural circuits and brain network connectivity in migraine and whether this parallels changes associated with ELS in the context of MDD.

Preclinical studies of chronic stress in adult individuals have also been conducted with regard to both depressive and migraine-related phenotypes. With regard to depression, several models of chronic stress have emerged as having face, construct, and predictive validity to the human condition [317, 318]. These include chronic social defeat stress (CSDS) and chronic unpredictable stress (CUS) [319, 320]. Studies using these paradigms have identified brain-wide neural circuit connectivity changes and brain-wide transcriptional regulations between animals that are resilient and those that are susceptible to a depressive syndrome following stress and largely parallel human findings regarding MDD and brain network activity [226, 321–324]. From these studies, we know that prefrontal cortex (PFC) connectivity to limbic regions such as amygdala, ventral hippocampus, and especially reward-related limbic regions such as nucleus accumbens (NAc) and ventral tegmental area (VTA) define the difference between susceptibility and resilience. Interestingly, CSDS has also been shown to have an impact on vulnerability to migraine-like phenotypes in a mouse model of migraine as well [325]. Using NTG to induce a migraine-like state characterized by allodynia and cortical spreading depression, Kaufmann & Brennan showed that depression-resilient mice showed a more rapid recovery from allodynia as measured by paw withdrawal, thus linking [325]. Notably, consistent with other characterizations of stress susceptibility in the CSDS paradigm, these differences were identified despite a lack of difference in cortisol levels between stress susceptible and resilient mice, further suggesting that the difference comes from downstream circuit-level differences and not at the level of general HPA-axis regulation. Another migraine-CSDS mechanistic link comes from studies of interleukin 6 (IL-6). IL-6 is an inflammatory cytokine that has been shown to both predict the emergence of a depression-susceptibility phenotype following CSDS [326] and sensitize mice and rats to the emergence of migraine-like phenotypes when applied to the dura [327, 328].

Further preclinical studies probing the relationship between stress and migraine-like phenotypes in adults make use of other stress paradigms such as repetitive restraint stress, unpleasant sensory stimuli, predators, and other unpredictable stressors, sometimes in various combinations. Paradigms very closely paralleling the chronic unpredictable stress paradigm used to study depression circuits have been used with genetic models of migraine susceptibility such as that of familial hemiplegic migraine (FHM) [329]. Interestingly, relief from such chronic stress, rather than the stress itself was found to exacerbate cortical spreading depression in FHM mice, paralleling many human patient reports of migraine after a stressor is over [329]. Related studies strongly implicate adrenergic neural circuits, which play an important role in the development of depression as well as migraine (as discussed above) [242, 330, 331]. Unpredictable sound stress model causes migraine-like and depressive-like behaviors in rodents abolished by CGRP receptor antagonist [332]. Chronic unpredictable mild stress was shown to lead to a shared pain (thermal sensitivity) and depression phenotype in mice, which could be negatively regulated with D2-dopmaine receptor agonism [333]. A preclinical study seeking to dive more deeply into the sex × stress clinical observations interrogated the relationship between the hormone prolactin and migraine response. This study found that repeated restraint stress increased prolactin levels in female mice, resulting in increased facial hypersensitivity [334]. Disrupting prolactin signaling in sensory neurons attenuates facial hypersensitivity in female, but not male mice, suggesting yet another female-specific mechanism by which stress exacerbates migraine-like phenotypes [334]. Interestingly, in humans plasma, prolactin is higher in patients with major depressive disorder compared with controls and higher in women than men [335].

Although some of the mechanisms we’ve discussed in this section so far occur independently of the HPA-axis, HPA-axis engagement and its regulation also plays an important role in migraine and depression. The HPA-axis regulates the release of glucocorticoids and there is evidence of elevated cortisol levels under certain conditions in both migraine and depression, suggesting maladaptive stress responses being an integral part of both disorders [336, 337]. In migraine, elevated cortisol levels in the serum and CSF have been found to be higher in chronic migraine patients compared to episodic migraine and healthy control patients, which suggests a possible role of glucocorticoid biomarkers in migraine chronification [336]. However, there were several limitations in this particular study including the use of migraine medications like sumatriptan which could alter monoamine levels, as well as the timing of serum and CSF collection. In a preclinical model, corticosterone was shown to induce migraine-like behaviors specifically in female mice while the glucocorticoid antagonist mifepristone blocked stress-induced migraine-like behaviors in both male and female mice [338]. This study provides an avenue to pin-pointing specific physiological mechanisms of HPA-axis dependent migraine from amidst the wide-range of glucocorticoid-induced mechanisms. In MDD, extensive studies have investigated a role for HPA-axis in the development of pathology relating to its impact on neuroplasticity, neurotransmitter systems, immune response, metabolism, oxidation, and gut microbiota [284, 339–341]. The relationship of cortisol regulation to MDD specifically is complex and can depend on life stage, phase, and severity of the disorder, as well as whether baseline or challenge levels of are being measured [337]. However, elevated baseline and challenge serum and saliva cortisol levels have been linked to severity of MDD, especially what is considered more severe subtypes such as psychotic and melancholic MDD [337]. Overall, numerous findings support the involvement of HPA-axis regulation in both disorders, but also suggest distinctions in the conditions of the specific ways that the HPA-axis is disrupted in each disorder. Mechanistically, a possible link between HPA-axis regulation, sleep, headache, and depression is brain glycogen metabolism [342]. Sleep disturbances and stress can disrupt glycogen turnover, potentially impairing energy supply to glutamatergic neurons, which may contribute to worsening mood symptoms and vulnerability to headaches [342, 343].

As noted in the pharmacology and treatment section, PACAP also interacts with glucocorticoids in ways that influence both mood and migraine-like responses. Following CSDS, PACAP-deficient mice exhibit significantly reduced serum corticosterone (CORT) levels, along with decreased FosB-positive cells in the paraventricular nucleus of the hypothalamus (PVN) and increased FosB expression in the medial prefrontal cortex (mPFC) [87]. These findings suggest that PACAP promotes stress-induced neuroendocrine and neural responses associated with depressive-like behavior. These results are also consistent with experiments involving intracerebroventricular (ICV) administration of PACAP in rodents, where stress-induced depressive-like behaviors have been observed [95]. Overall, the role of PACAP in HPA-axis dysregulation may simultaneously promote long-term maladaptive plasticity in the brain biasing towards negative affective states as well as peripheral changes that increase the susceptibility of migraine attack initiation [97]. Future PACAP-related therapeutics may benefit from monitoring of glucocorticoid levels, especially when considering patients with the comorbidity.

Endogenous opioids are released in response to stress, and can regulate, augment, attenuate or prevent both migraine and MDD depending on the duration and context of exposure [344–347]. A study in a rodent model of repetitive restraint stress demonstrated that the endogenous opioid enkephalin is capable of blocking stress-induced migraine hypersensitivity [348]. Alternatively, the endogenous opioid dynorphin exacerbated trigeminal pain in a mouse model of migraine and paralleled findings for chronic stress induction of depression [344, 345]. Furthermore, the long and short-term impacts of exercise on both depression and migraine suggest a role for endogenous opioids and neurotrophic factors (e.g. brain-derived neurotrophic factor, BDNF). Exercise is known to increase BDNF levels and thus contribute to the prevention of neuronal loss and improved cognitive functioning, and, in the case of depression, it also demonstrates protective effects [349, 350]. Examination of BDNF levels following exercise in migraine patients still needs to be explored, however, serum BDNF levels tend to be higher during migraine attacks, which may contribute to the complex relationship between migraine and exercise [351, 352]. Exercise can play a role in the management of both disorders, however, that relationship is complex. Symptoms of both migraine and MDD have been shown to be prevented by exercise, though meta-analysis studies show a large heterogeneity of responses and that exercise can also exacerbate migraine under certain conditions [308, 351, 353].

Finally, it’s worth revisiting possible mechanisms of sex differences with regard to stress and the HPA-axis. Estrogen has an influence on neurotransmitter systems implicated in both disorders (serotonin, dopamine, and glutamate), stress reactivity (by the HPA axis, corticotrophin-releasing hormone), neurotrophic factors and neuropeptides like neuropeptide Y, and cortical excitability which all contribute to both migraine and mood regulation [354]. Fluctuations in estrogen, particularly during the menstrual cycle, postpartum period, or menopause, can destabilize these pathways and may contribute to the disproportionately high rates of comorbidity between MDD and migraine in women [125, 126, 354, 355]. Progesterone is also a candidate for mechanisms explaining the comorbidity, as its role in depression is complex and understudied, and recent preclinical studies indicate its role in predisposition to cortical spreading depression as that experienced in migraine [356, 357].

### Limitations

This is a narrative, not systematic, review. We as authors did our best to survey the literature in a logical way, spanning levels of analysis, however, identifying mechanisms that are common across fields does require some pre-existing understanding of each field, and, as such, is subject human error. Furthermore, there is some subjectivity in the interpretation of what data can or should reasonably be called “mechanistic”. The Oxford English dictionary defines mechanism as, “a natural or established process by which something takes place or is brought about.” We focused on the latter part of this definition, choosing to emphasize shared factors that to us most suggested possible shared causality. To us, the emphasis on mechanisms necessitated dedicating a substantial portion of the work reported to preclinical studies, as these are often the studies where variables can best be controlled under laboratory conditions. Nevertheless, we fully acknowledge that in doing so, there may be some important topics left out. Readers interested in more clinical and epidemiological features shared between these populations may find value in several other helpful reviews [5, 358, 359].

Another limitation is that we did not include a specific section on lifestyle. Instead, we acknowledged aspects of lifestyle contributions in different sections, highlighting mechanisms that are influenced by lifestyle factors such as sleep (NE/LC function, HPA-axis regulation), exercise (BDNF expression, endogenous opioid role, ELS), and diet (gut microbiome, vitamin B2 intake). In addition to the references provided above regarding clinical findings that overlap between MDD and migraine, several additional recent studies may be of interest on this topic demonstrating the impact of environmental exposures, psychotherapy, mindfulness and leisure time on the comorbid migraine and MDD condition [360–366].

Also, as mentioned in the introduction, the underlying mechanisms of both of these disorders is still a matter of much study and debate. While progress has been made, there is still a lot of work that needs to be done. This review provides only a snapshot of the mechanisms common to both disorders, limited by each field’s current understanding of the factors that are most likely causal. As each field grows and evolves, the features for which the most shared mechanistic overlap will no doubt change as well. Finally, although we discuss comorbidity, we report on many studies that evaluated depression and migraine separately and looked for common mechanistic contributors. We deemed this necessary at this stage of research where even clinical studies are limited and preclinical studies of the comorbid condition have largely not been developed yet. We hope that in doing so, this review will inspire research that will shed light on the mechanisms of the comorbid condition.

## Conclusions

**Fig. 1:**
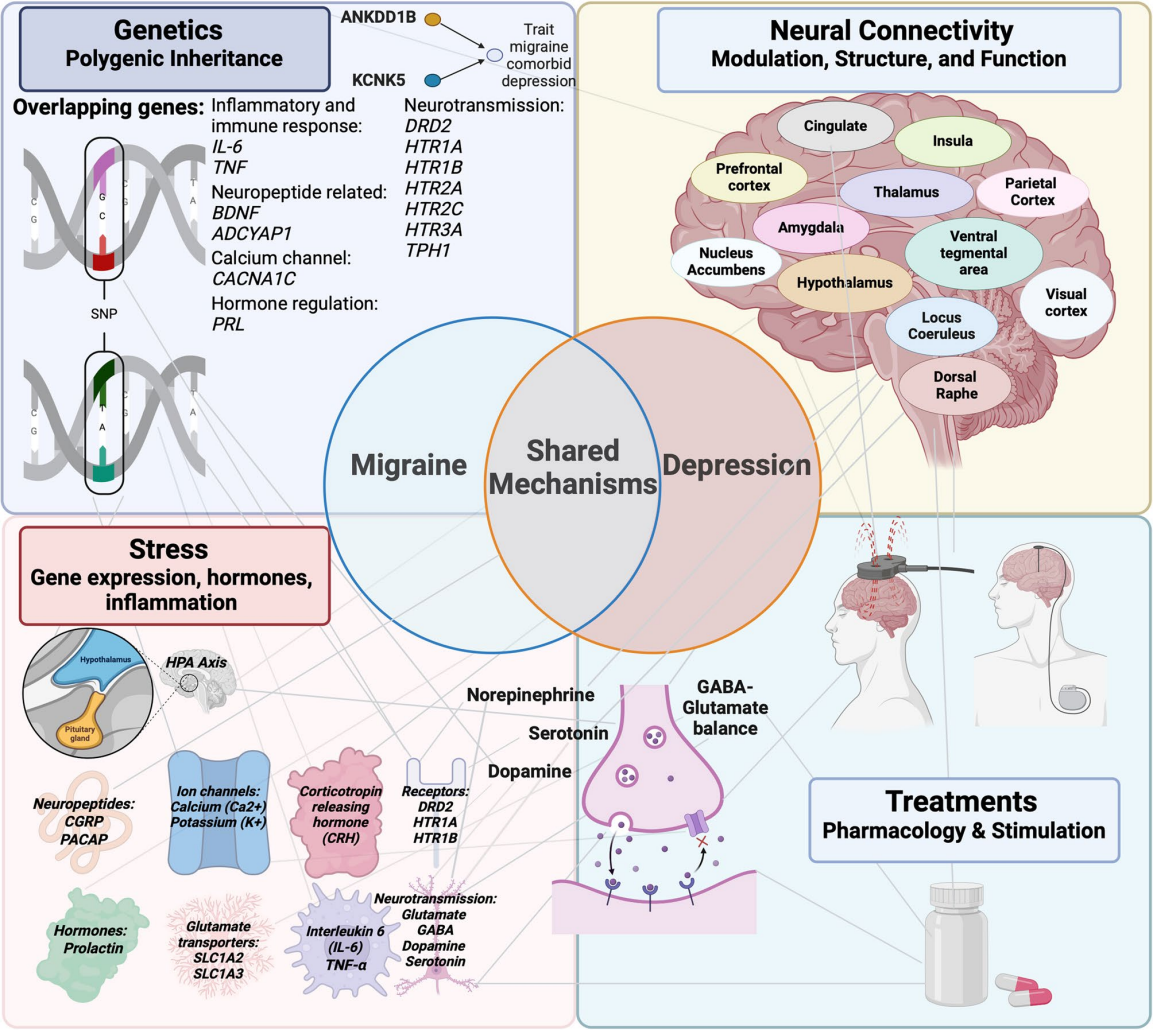
Shared mechanisms between migraine and MDD across neurotransmitter/neuromodulator systems (y-axis) and across pharmacological treatments, neural connectivity, and peripheral mechanisms (x-axis)

**Fig. 2:**
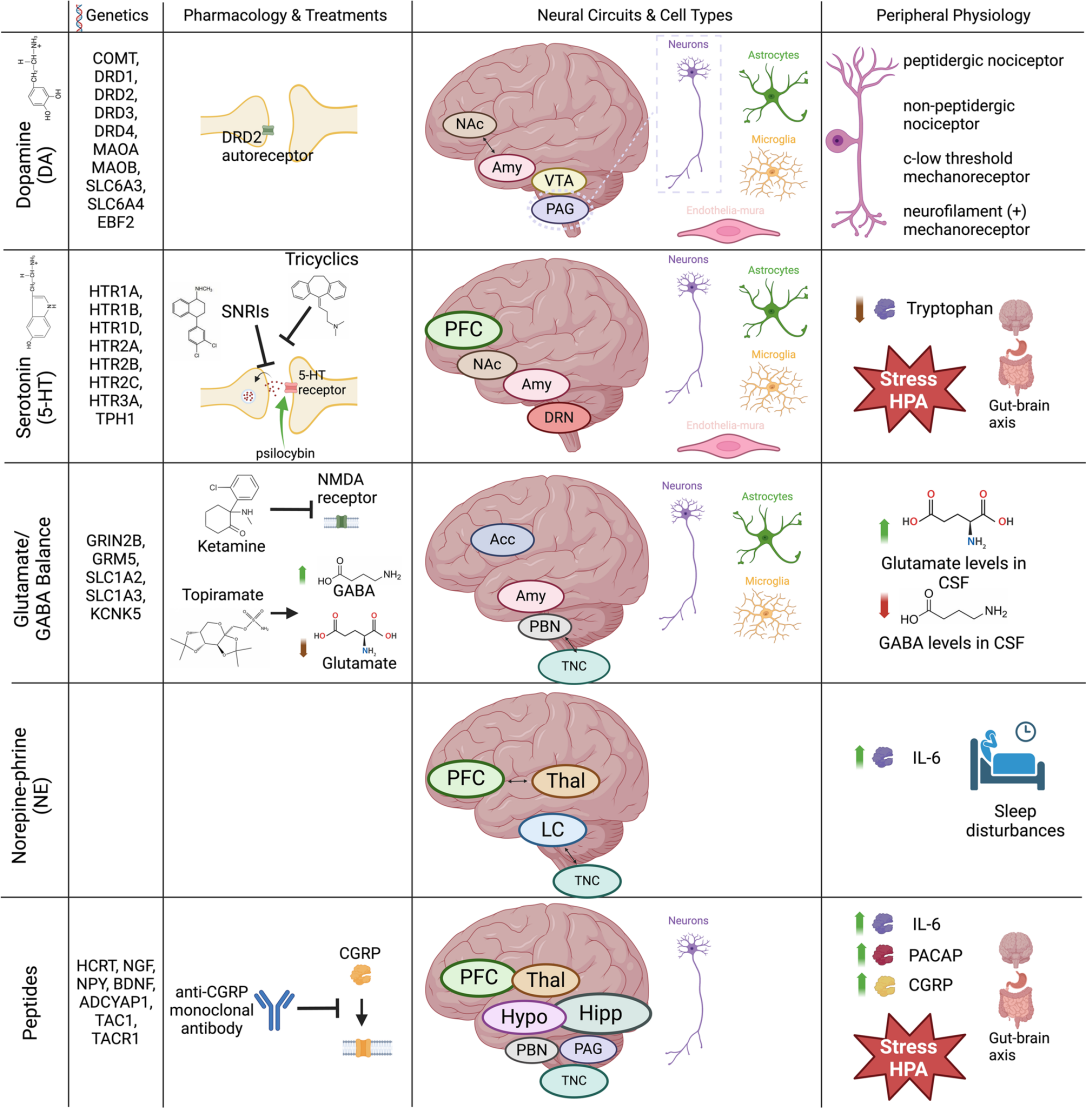
Integration of mechanisms spanning genes, molecules, synapses, neural circuits, and environmental contributors

Here we have reviewed much of the evidence for mechanistic interplay between the underlying etiology of two disorders for which the root causes are still not definitively elucidated (Fig. 1). We have especially sought to emphasize preclinical models, as they have enabled mechanistic causality studies. From these studies, it is evident that migraine and MDD share common mechanistic threads across genetic, molecular, neural circuit, and environmental response levels (Fig. 2). We hope that this review of shared mechanisms between migraine and MDD will ignite more conversation, attention, and research around the potential therapeutic importance of this comorbidity, beyond a common attribution of physical pain causing emotional pain. For clinicians, we hope that providing detailed mechanistic relationships between these disorders may inspire fresh insights into the range of possible therapeutic considerations for patients with this comorbidity. It is our view that conducting further studies in migraine models evaluating MDD-related mechanisms and vice versa are likely to shed light on common mechanisms underlying both disorders. Furthermore, adding metrics that probe constructs related to both disorders (e.g. sensory hypersensitivity assays in depression models and cortico-limbic affective circuits and neuromodulators in migraine models), will likely promote better understanding and classification of the underlying mechanisms of each disorder.

## Supplementary Information

Supplementary Material 1: Table S1. Studies in humans and rodents providing evidence of role of PACAP in migraine and MDD. This table summarizes key papers from clinical and preclinical data indicating a role for PACAP in MDD and migraine. Papers focused on human data is in darker colors with MDD-related papers in blue and migraine-related papers in orange. Where a large number of papers were found to demonstrate similar results, we have included those that provide sufficient differences in the conditions that provide insight into the different conditions by which PACAP influences disease and related behaviors, prioritizing papers with both sexes wherever possible and those with higher n and multiple metrics

## Data Availability

No datasets were generated or analysed during the current study.

## Abbreviations

MDD: Major depressive disorder
MWA: Migraine with aura
DSM: Diagnostic and Statistical Manual
ELS: Early life stress
FHM: Familial hemiplegic migraine
NTG: Nitroglycerin
GWAS: Genome wide association study
CNS: Central Nervous System
CSDS: Chronic social defeat stress
CUS: Chronic unpredictable stress
FHM: Familial hemiplegic migraine
LC: Locus coeruleus
VTA: Ventral tegmental area
DR: Dorsal raphe nucleus
PNS: Peripheral Nervous System
PVN: Paraventricular nucleus of the hypothalamus
TCC: Trigeminal cervical complex
ACC: Anterior cingulate cortex
vPAL: Ventral pallidum
NAc: Nucleus accumbens
PFC: Prefrontal cortex
dlPFC: Dorsolateral prefrontal cortex
mPFC: Medial prefrontal cortex
VTA: Ventral tegmental area
Sp5C: Spinal trigeminal subnucleus caudalis
LPBN: Lateral parabrachial nucleus
TCAs: Tricyclic antidepressants
SNP: Single nucleotide polymorphism
SSRIs: Selective serotonin reuptake inhibitors
SNRIs: Serotonin-norepinephrine reuptake inhibitors
DBS: Deep brain stimulation
5-HT: Serotonin
DRD2: Dopamine D2 receptor
SERT: Serotonin transporter
CSD: Cortical spreading depression
CGRP: Calcitonin gene-related peptide
NET: Norepinephrine transporter
NPY: Neuropeptide Y
PACAP: Pituitaryadenylate cyclase-activating polypeptide
NMDA: N-methyl-D-aspartate
CSF: Cerebrospinal fluid
GABA: γ-aminobutyric acid
BDep: Bipolar depression
HCN: Hyperpolarization-activated cyclic nucleotide-gated
IL-6: Interleukin-6
PET: Positron emission tomography
EEG: Electroencephalography
fMRI: Functional magnetic resonance imaging
TMS: Transcranial magnetic stimulation
TCS: Transcranial sonography
GI: Gastrointestinal
HPA: Hypothalamo-pituitary axis
VIP: Vasoactive intestinal polypeptide
Sst: Somatostatin
CRF: Corticotropin-releasing factor
SCFAs: Short chain fatty acids
IBS: Irritable bowel syndrome
BDNF: Brain-derived neurotrophic factor
